# Appropriate Drought Training Induces Optimal Drought Tolerance by Inducing Stepwise H_2_O_2_ Homeostasis in Soybean

**DOI:** 10.3390/plants13091202

**Published:** 2024-04-25

**Authors:** Yuqian Shen, Lei Li, Peng Du, Xinghua Xing, Zhiwei Gu, Zhiming Yu, Yujia Tao, Haidong Jiang

**Affiliations:** 1 Key Laboratory of Crop Physiology and Ecology in Southern China, Ministry of Agriculture and Rural Affairs, Jiangsu Collaborative Innovation Center for Modern Crop Production, Nanjing Agriculture University, Nanjing 210095, China; 2021101022@stu.njau.edu.cn (Y.S.); ll18051507978@163.com (L.L.); dp19920818@163.com (P.D.); 20231079@qhnu.edu.cn (Z.G.); 17761615319@163.com (Z.Y.); 18705190108@139.com (Y.T.); 2 Xuzhou Institute of Agricultural Sciences of Xu-Huai Region of Jiangsu, Xuzhou 221131, China

**Keywords:** soybean, H_2_O_2_ homeostasis, ROS, antioxidant system, PEG-simulated drought stress, signaling

## Abstract

Soybean is considered one of the most drought-sensitive crops, and ROS homeostasis can regulate drought tolerance in these plants. Understanding the mechanism of H_2_O_2_ homeostasis and its regulatory effect on drought stress is important for improving drought tolerance in soybean. We used different concentrations of polyethylene glycol (PEG) solutions to simulate the progression from weak drought stress (0.2%, 0.5%, and 1% PEG) to strong drought stress (5% PEG). We investigated the responses of the soybean plant phenotype, ROS level, injury severity, antioxidant system, etc., to different weak drought stresses and subsequent strong drought stresses. The results show that drought-treated plants accumulated H_2_O_2_ for signaling and exhibited drought tolerance under the following stronger drought stress, among which the 0.5% PEG treatment had the greatest effect. Under the optimal treatment, there was qualitatively describable H_2_O_2_ homeostasis, characterized by a consistent increasing amplitude in H_2_O_2_ content compared with CK. The H_2_O_2_ signal formed under the optimum treatment induced the capacity of the antioxidant system to remove excess H_2_O_2_ to form a primary H_2_O_2_ homeostasis. The primary H_2_O_2_ homeostasis further induced senior H_2_O_2_ homeostasis under the following strong drought and maximized the improvement of drought tolerance. These findings might suggest that gradual drought training could result in stepwise H_2_O_2_ homeostasis to continuously improve drought tolerance.

## 1. Introduction

Soybean (*Glycine max*) is one of the major sources of plant-based protein and oil for human consumption. Over the past decade, most research in the agronomy section has emphasized understanding the effect of such deviations in abiotic stresses on different plant species, such as soybean [1] and wheat plant growth and production, which can cause stress, such as heat [2], salt [3], drought, and water deficit [4]. Soybean plants have high water requirements for growth and underdeveloped root systems, so they are extremely susceptible to drought [5]. Drought is the major limiting factor for soybean production, and drought alone causes more annual losses in crop yield than all the other stresses combined [6].

ROS are highly active but are normally limited to a certain level to prevent unintended cellular oxidation [7]. However, drought stress can alter metabolic pathways in plants, leading to ROS accumulation. ROS are toxic and can cause oxidative stress [8,9]. Moreover, ROS function as signaling molecules that are capable of influencing the expression of many genes via a large number of signal transduction pathways that activate and control various stress programs in organisms [10]. Whether ROS act as signaling molecules or produce oxidative stress injury to the plant depends on the site, time, and concentration of the reactive oxygen species produced [11].

The concept of homeostasis was proposed by Claude Bernard as early as the 19th century [12]. It refers to the state in which the internal environment of an organism remains relatively stable. Homeostasis allows biological systems to make continuous short-term self-adjustments for optimal functioning while changing internal and external environments. It can be used in organisms, such as humans, higher animals, plants, and cells. In plants, many kinds of homeostasis, such as zinc homeostasis [13], sugar homeostasis [14], ABA homeostasis [15], and ROS homeostasis [16], have been studied. The baseline level of ROS is a characteristic of cellular homeostasis and can be disrupted by abiotic stresses [7]. Disrupted cellular homeostasis can be regulated by redox balance and ion homeostasis [17]. ROS are currently recognized as key signals corresponding to plant stress. To reveal their signaling mechanism and characterize their relevant components, in-depth research on ROS homeostasis is needed [18].

H_2_O_2_ is one of the ROS with the highest stability and longest survival time. It is easy for this material to passively pass through membranes and/or water channels. It is well suited for transmitting signals between cells. Thus, H_2_O_2_ is the most studied signaling molecule [19]. Although many reports agree that there is a balance between the scavenging and the production of H_2_O_2_ in plants, this balance has been described only qualitatively rather than quantitatively [19,20,21,22,23,24]. Previous studies have suggested that stress tolerance can be only modulated by regulating ROS homeostasis. Many studies have only focused on studying the changes between treatment onset and endpoints rather than the entire treatment period [25,26], which involves a greater adaptive range in response to stress. Adaptive homeostasis is activated to signal when external or internal perturbations exceed the homeostatic range. Since the adaptive response is transient, after a period of time, adaptive homeostasis in animals gradually changes back to the initial basal homeostatic range [12,27]. However, unlike animals, plants cannot escape stress and can only passively respond and adapt. Under sustained, stable, and weak stress, plants exhibit decreased cell damage, easily controlled ROS production, and increased survival time [28], which suggests that there might be a temporary increase in the homeostasis of ROS during treatment compared with that during normal homeostasis. This temporary homeostasis can be manifested by comparing the changing amplitude of ROS between normal and stressed conditions during a period of treatment. Moreover, measurable homeostasis can help reveal the mechanism underlying the formation and function of ROS homeostasis.

Drought training is carried out mainly under weak drought stress. During progressive soil drying, ROS homeostasis acts as a regulator in relationships between the soil–water threshold range of chemical signals and drought tolerance in wheat [29]. Therefore, plants may maintain temporary ROS homeostasis to adapt to sustained weak drought stress. Therefore, we subjected soybean seedlings to different levels of weak and strong drought stress conditions to determine whether H_2_O_2_ homeostasis may occur in the best-performing plants. We investigated the phenotype, ROS levels, injury severity, and antioxidant system response to different treatments to clarify the specific manifestations of H_2_O_2_ homeostasis and reveal the mechanism and function of H_2_O_2_ homeostasis. This study may help to further understand H_2_O_2_ homeostasis.

## 2. Results

### 2.1. Leaf Morphology

**Figure 1:**
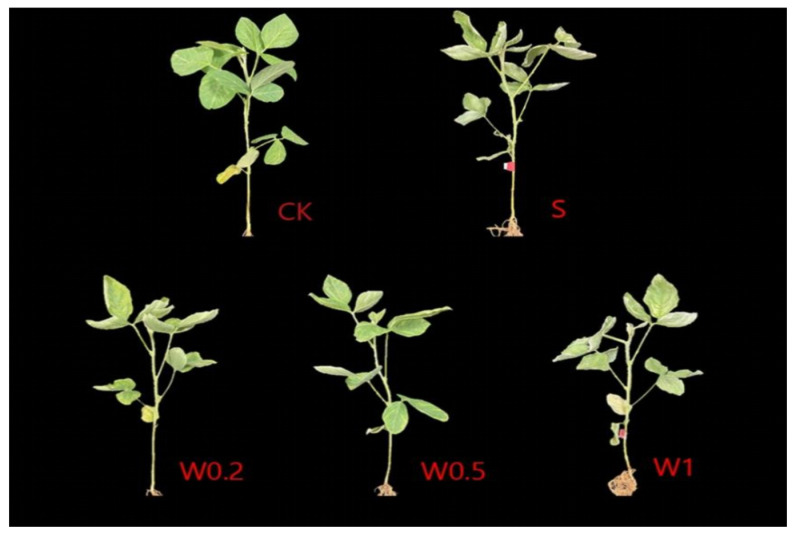
Phenotypic changes of “Kefeng 1” soybean under different treatments at 24 h after PEG-simulated drought treatment. Among them, CK: hydroponic Hoagland nutrient solution; W0.2, W0.5, and W1: 0.2%, 0.5%, and 1% PEG Hoagland nutrient solution, respectively, that was then transferred to 5% PEG Hoagland nutrient solution; and S: hydroponic growth of the Hoagland nutrient solution for 6 h, followed by transfer to 5% PEG Hoagland nutrient solution.

The leaves of the soybean seedlings cultured under normal conditions spread. Compared with those in the CK treatment, the leaves of the soybean seedlings in the W0.2 and W1 treatments were significantly curled at 24 h, while those in the W0.5 treatment exhibited the slightest curling at 24 h. In contrast, the leaves of the soybean seedlings in the S treatment were significantly curled and yellowed at 24 h, indicating the most severe injury (Figure 1).

### 2.2. Leaf Water Content

**Figure 2:**
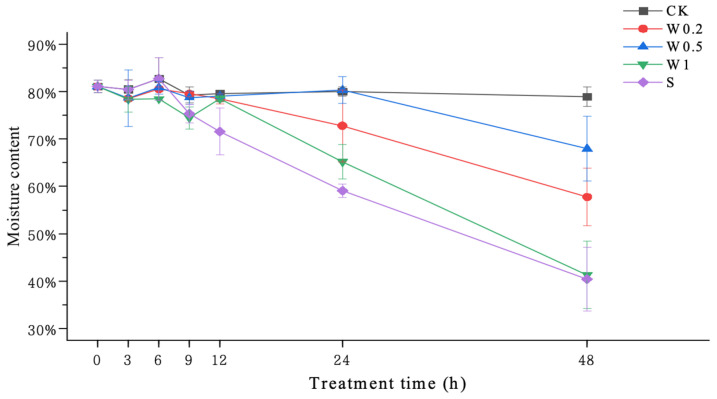
Changes in the moisture content of “Kefeng 1” soybean leaves under different treatments at 0 h, 3 h, 6 h, 9 h, 12 h, 24 h, and 48 h after PEG-simulated drought treatment. Among them, CK: hydroponic Hoagland nutrient solution; W0.2, W0.5, and W1: 0.2%, 0.5%, and 1% PEG Hoagland nutrient solution, respectively, that was then transferred to 5% PEG Hoagland nutrient solution; and S: hydroponic growth of the Hoagland nutrient solution for 6 h, followed by transfer to 5% PEG Hoagland nutrient solution. The error bars represent the SDs of means.

The water content of the soybean leaves under CK fluctuated slightly during the treatment. Under weak drought stress, the water content of the soybean leaves in the W0.2, W0.5, and W1 treatments first decreased and then increased. Upon entering strong drought stress, the water content of the soybean leaves treated with W0.5 did not change much during the 6–24 h period, and it was not significantly different from that in the CK treatment, although it did not significantly decrease beyond 24 h with increasing time. The water content of the soybean leaves treated with W1 showed a decreasing trend within 6–9 h, an increasing and then decreasing trend during 9–48 h, and a significant decrease at 48 h. The water content of the soybean leaves in the W0.2 and S treatments showed a consistent decreasing trend and decreased significantly at 48 h (Figure 2).

### 2.3. Antioxidant System

#### 2.3.1. MDA Content

The MDA content of the soybean leaves cultured in the CK treatment showed a constant increasing trend. Under weak drought stress, the W0.2, W0.5, and W1 treatments all presented a decreasing and then increasing trend within 6 h. At 6 h, the MDA content in the W0.5 treatment was basically the same as that in the CK treatment.

**Figure 3:**
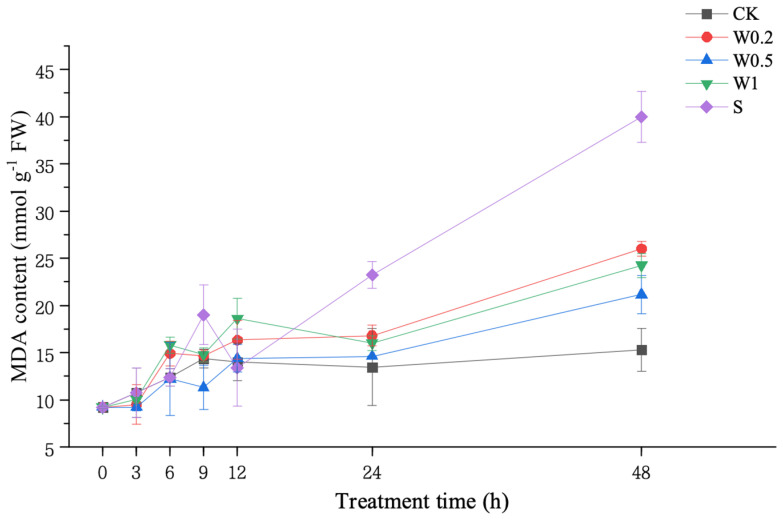
Changes in the MDA content of “Kefeng 1” soybean leaves under different treatments at 0 h, 3 h, 6 h, 9 h, 12 h, 24 h, and 48 h after PEG-simulated drought treatment. Among them, CK: hydroponic Hoagland nutrient solution; W0.2, W0.5, and W1: 0.2%, 0.5%, and 1% PEG Hoagland nutrient solution, respectively, that was then transferred to 5% PEG Hoagland nutrient solution; and S: hydroponic growth of the Hoagland nutrient solution for 6 h, followed by transfer to 5% PEG Hoagland nutrient solution. The error bars represent the SDs of means.

Upon entering strong drought stress, the MDA content of soybean leaves in the W0.2, W0.5, and W1 treatments all decreased and then increased during 6–12 h, which was consistent with the trend within 6 h. At 48 h, the W0.2 and W1 treatments were significantly higher than that of CK, with the lowest increase in the MDA content in the W0.5 treatment. However, the MDA content of the soybean leaves in the S treatment was significantly higher than that in the CK treatment at 48 h (Figure 3).

#### 2.3.2. H_2_O_2_ Content and O_2_^−^ Production Rate

**Figure 4:**
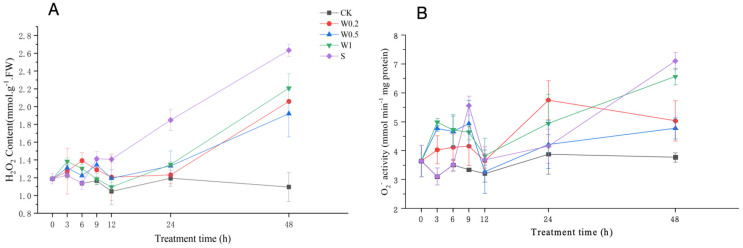
H_2_O_2_ content (**A**) and O_2_^−^ production rate (**B**) of “Kefeng 1” soybean leaves under different treatments at 0 h, 3 h, 6 h, 9 h, 12 h, 24 h, and 48 h after PEG-simulated drought treatment. Among them, CK: hydroponic Hoagland nutrient solution; W0.2, W0.5, and W1: 0.2%, 0.5%, and 1% PEG Hoagland nutrient solution, respectively, that was then transferred to 5% PEG Hoagland nutrient solution; and S: hydroponic growth of the Hoagland nutrient solution for 6 h, followed by transfer to 5% PEG Hoagland nutrient solution. The error bars represent the SDs of means.

The H_2_O_2_ content of the leaves of soybean plants cultured in the CK treatment fluctuated with time. Under weak drought stress, the H_2_O_2_ content in the W0.2 treatment increased within 6 h, while it first increased and then decreased in the W0.5 and W1 treatments. Upon entering strong drought stress, the H_2_O_2_ content of soybean leaves treated with W0.5 decreased and then increased during 9–48 h. The H_2_O_2_ content in both the W0.2 and W1 treatments decreased briefly and then increased during 6–48 h and was significantly higher than that in the CK treatment at 48 h. The H_2_O_2_ content in the S treatment increased rapidly and was also significantly higher than that in CK at 48 h. Notably, compared with that in the CK treatment, the H_2_O_2_ content in the W0.5 treatment increased by 7.2% and 7.3% at 3 h and 6 h, respectively, after weak stress. Upon entering strong stress, the H_2_O_2_ content increased by 15.4%, 14.1%, and 14.5%, respectively, compared with that in the CK group at 9 h, 12 h, and 24 h. As shown in the figure, we can clearly find that the magnitude of change in the H_2_O_2_ content in the W0.5 treatment group at 3–6 h, 9–12 h, and 12–24 h was basically the same as that in the CK group at the corresponding time points (Figure 4A).

The O_2_^−^ production rate of soybean leaves in the CK treatment showed a decreasing and then increasing trend. Under weak drought stress, the O_2_^−^ production rate of the soybean leaves in the W0.5 and W1 treatments first increased and then decreased, while it increased continuously in the W0.2 treatment. Upon entering strong drought stress, the O_2_^−^ production rate of soybean leaves in the W0.2 and W0.5 treatments first increased and then decreased during 6–12 h. With the extension of strong drought stress, the O_2_^−^ production rate increased substantially during 12–48 h and was significantly higher than that of CK at 48 h. This effect was basically the same for W0.5 at 12 h. Similarly, the O_2_^−^ production rate of the soybean leaves in the W1 and S treatments was significantly higher than that in the CK treatment at 48 h (Figure 4B).

#### 2.3.3. SOD, POD, and CAT Activities

**Figure 5:**
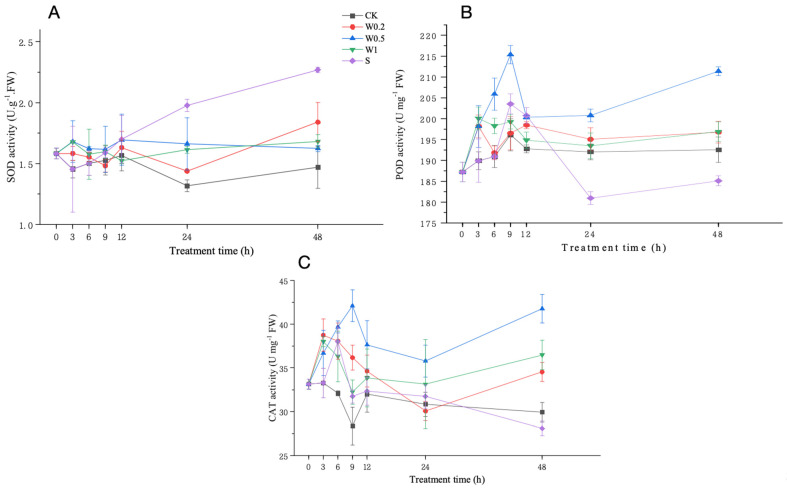
Changes in the activities of SOD (**A**), POD (**B**), and CAT (**C**) of “Kefeng 1” soybean leaves under different treatments at 0 h, 3 h, 6 h, 9 h, 12 h, 24 h, and 48 h after PEG-simulated drought treatment. Among them, CK: hydroponic Hoagland nutrient solution; W0.2, W0.5, and W1: 0.2%, 0.5%, and 1% PEG Hoagland nutrient solution, respectively, that was then transferred to 5% PEG Hoagland nutrient solution; and S: hydroponic growth of the Hoagland nutrient solution for 6 h, followed by transfer to 5% PEG Hoagland nutrient solution. The error bars represent the SDs of means.

Under weak drought stress, the SOD activity of soybean seedlings cultured in the CK treatment first decreased and then increased, while it first increased and then decreased within 6 h in the W0.2, W0.5, and W1 treatments, and it experienced the most increase in the W0.5 treatment compared with the CK treatment. Upon entering strong drought stress, the SOD activity of soybean leaves treated with W0.2 first decreased and then increased during 6–12 h, while it first decreased and then increased after 12 h and was significantly higher than that in CK at 48 h. The SOD activity of soybean leaves treated with W0.5 first decreased and then increased during 6–12 h. From 12–48 h, the SOD activity gradually decreased and was significantly higher than that in the CK group at 48 h. The SOD activity in the W1-treated leaves first decreased and then increased during the 6–48 h period and was significantly higher than that in the CK group at 48 h. However, the SOD activity in the S-treated leaves consistently increased and was significantly higher than that in the CK leaves at 48 h (Figure 5A).

Under weak drought stress, the POD activity in the soybean leaves treated with the W0.2 or W1 treatments first increased but subsequently decreased, while it consistently increased in the W0.5 treatment and was significantly higher than that in the CK treatment at 6 h. Upon entering strong drought stress, the POD activity in the W0.2, W0.5, and W1 treatments increased and then decreased during 6–12 h. As the duration of strong drought stress increased, the POD activity of the W0.5 treatment increased significantly more than that in CK at 48 h. Similarly, the POD activity in the S treatment increased first, then decreased, and finally increased during 6–48 h and was significantly lower than that in CK at 48 h (Figure 5B).

Under weak drought stress, the CAT activity in the W0.2 and W1 treatments first increased and then decreased, while it showed a consistent increasing trend in the W0.5 treatment. Upon entering strong stress, the CAT activity in the W0.2 and W1 treatments exhibited a decreasing trend in the first 24 h and then an increasing trend. In contrast, the CAT activity in the W0.5 treatment peaked at 9 h and displayed a second increase after 24 h. The CAT activity in the S treatment first increased and then decreased and was lower than that in the CK treatment at 48 h (Figure 5C).

#### 2.3.4. ASA and GSH Contents

**Figure 6:**
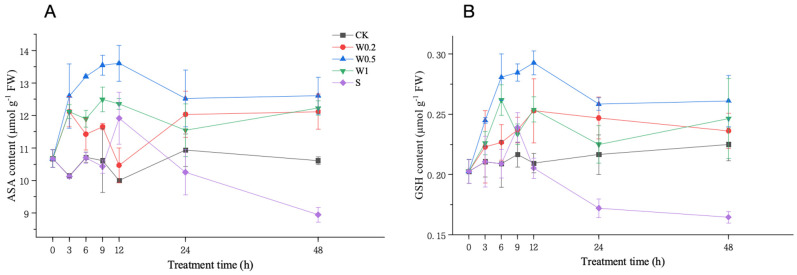
Changes in the ASA (**A**) and GSH (**B**) contents of “Kefeng 1” soybean leaves under different treatments at 0 h, 3 h, 6 h, 9 h, 12 h, 24 h, and 48 h after PEG-simulated drought treatment. Among them, CK: hydroponic Hoagland nutrient solution; W0.2, W0.5, and W1: 0.2%, 0.5%, and 1% PEG Hoagland nutrient solution, respectively, that was then transferred to 5% PEG Hoagland nutrient solution; and S: hydroponic growth of the Hoagland nutrient solution for 6 h, followed by transfer to 5% PEG Hoagland nutrient solution. The error bars represent the SDs of means.

Under weak drought stress, the ASA content in the W0.2- and W1-treated leaves first increased and then decreased, and the ASA content of the W1 treatment was significantly higher than that in the CK treatment at 6 h. However, the ASA content in the W0.5 treatment consistently increased and was markedly greater than that in the CK treatment at 6 h. Upon entering strong drought stress, the ASA content in the W0.2, W0.5, and W1 treatments all increased first, decreased, and then increased after 6 h and was significantly higher than that in the CK treatment at 48 h in all three treatment groups. The ASA content in the S treatment group briefly decreased and then increased considerably during 6–12 h, followed by a decreasing trend after 12 h and was significantly lower than that in the CK group at 48 h (Figure 6A).

Under weak drought stress, the GSH content of the soybean leaves in the W0.5 and W1 treatment groups increased significantly more than that in the CK group at 6 h. Upon entering strong drought stress, the GSH content of the soybean leaves in the W0.2 and W0.5 treatments first increased but subsequently decreased during the 6–24 h period, while it first decreased, subsequently increased, then decreased, and finally increased in the W1 treatment group. The GSH content in the S treatment group first increased briefly and then decreased significantly and was significantly lower than that in the CK group at 48 h (Figure 6B).

Similarly, the ASA and GSH contents in the leaves of soybean plants treated with W0.5 were higher than those in the other treatment groups during the treatment period. However, the ASA and GSH contents in the S treatment increased upon the plants entering the strong stress treatment and then rapidly decreased, reaching significantly lower levels than those in the CK treatment in the later stage.

#### 2.3.5. Activities of MDHAR, DHAR, and GR

**Figure 7:**
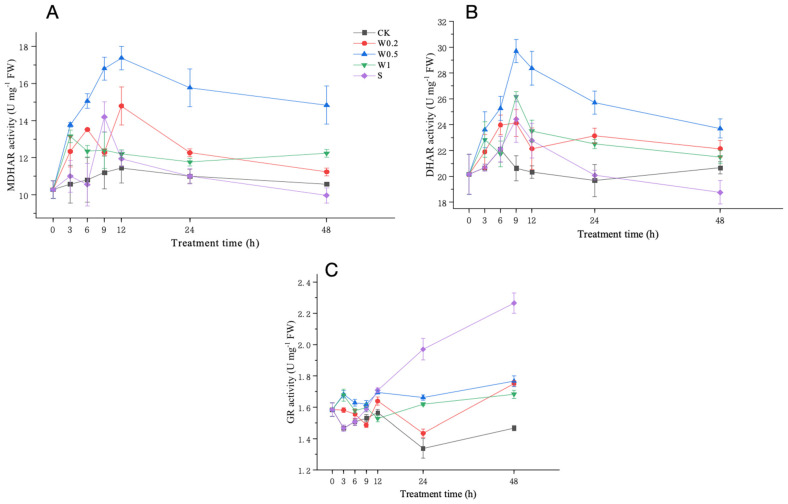
Changes in the MDHAR (**A**), DHAR (**B**), and GR (**C**) activities in “Kefeng 1” soybean leaves under different treatments at 0 h, 3 h, 6 h, 9 h, 12 h, 24 h, and 48 h after PEG-simulated drought treatment. Among them, CK: hydroponic Hoagland nutrient solution; W0.2, W0.5, and W1: 0.2%, 0.5%, and 1% PEG Hoagland nutrient solution, respectively, that was then transferred to 5% PEG Hoagland nutrient solution; and S: hydroponic growth of the Hoagland nutrient solution for 6 h, followed by transfer to 5% PEG Hoagland nutrient solution. The error bars represent the SDs of means.

Under weak drought treatment, the MDHAR activity in the W0.2- and W0.5-treated leaves consistently increased and was significantly higher than that in the CK leaves at 6 h. However, the MDHAR activity in the W1-treated plants first increased and then decreased. Upon entering strong drought stress, the MDHAR activity of the W0.2-treated leaves first briefly decreased, then increased, and finally decreased, and the activity level was higher than that of CK at 48 h. The MDHAR activity of the W0.5-treated leaves first increased and then decreased and was significantly higher than that of CK at 48 h. The MDHAR activity of the W1-treated leaves first increased and then decreased and was significantly higher than that of CK at 48 h. In contrast, the MDHAR activity of the S-treated leaves first briefly increased and then decreased and was lower than that of CK at 48 h (Figure 7A).

Under the weak drought treatment, the DHAR activity of soybean leaves cultured in a normal Hoagland nutrient solution did not change much. The DHAR activity in the W0.2 and W0.5 treatments consistently increased, while it first increased and then decreased in the W1 treatment. Upon entering strong drought stress, the DHAR activity in the W0.5 and W1 treatments first increased and then decreased after 6 h, and the DHAR activity of W0.5 was significantly higher than that in CK at 48 h. The DHAR activity in the W0.2 treatment first increased and then decreased during 6–12 h, followed by a trend of increasing first and then decreasing during 12–48 h. The DHAR activity in the S treatment first briefly increased and then decreased and was significantly lower than that in CK at 48 h (Figure 7B).

Under weak drought stress, the GR activity of soybean leaves treated with the W0.2, W0.5, and W1 treatments increased and then decreased, and the activity levels of the W0.5 and W1 treatments were significantly higher than that of CK at 6 h. Upon entering strong drought stress, the GR activity of soybean leaves treated with W0.5 and W1 decreased first, then increased, then decreased, and finally increased during 6–48 h. The GR activity of soybean leaves treated with W0.2 increased first and then decreased during 6–12 h, followed by an opposite trend during 12–48 h, and it was remarkably higher than that of CK at 48 h. The GR activity of the S-treated leaves increased the most. There was a consistent increasing trend upon entering strong drought, which was higher than that in CK at 48 h (*p* < 0.05) (Figure 7C).

#### 2.3.6. Activities of APX and GPX

**Figure 8:**
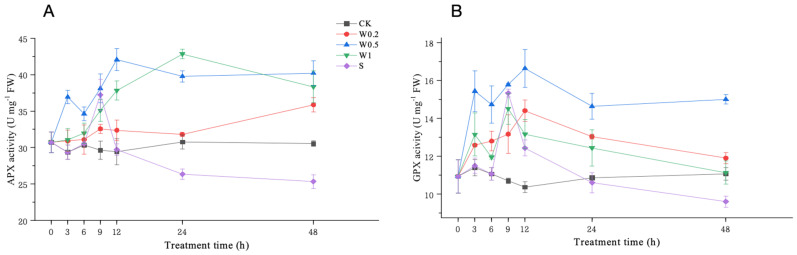
Changes in APX (**A**) and GPX (**B**) activities in “Kefeng 1” soybean leaves under different treatments at 0 h, 3 h, 6 h, 9 h, 12 h, 24 h, and 48 h after PEG-simulated drought treatment. Among them, CK: hydroponic Hoagland nutrient solution; W0.2, W0.5, and W1: 0.2%, 0.5%, and 1% PEG Hoagland nutrient solution, respectively, that was then transferred to 5% PEG Hoagland nutrient solution; and S: hydroponic growth of the Hoagland nutrient solution for 6 h, followed by transfer to 5% PEG Hoagland nutrient solution. The error bars represent the SDs of means.

Under weak drought stress, the APX activity in the soybean leaves treated with W0.2 or W1 increased slightly compared with that in the CK treatment, while it first increased rapidly and then decreased in the W0.5 treatment. Upon entering strong drought stress, the APX activity of the soybean leaves treated with W0.2 and W0.5 first increased and then decreased, followed by a consistent increasing trend and was significantly higher than that of CK at 48 h. The APX activity of the soybean leaves treated with W1 first increased and then decreased and was significantly higher than that of CK at 48 h. However, the APX activity of the S-treated leaves first increased rapidly, followed by a substantial decrease. It was significantly lower than that in CK at 48 h (Figure 8A).

Under weak drought stress, the GPX activity in the soybean leaves treated with W0.5 or W1 first increased and then decreased and was higher than that in the CK treatment at 6 h (*p* < 0.05). However, the GPX activity in the soybean leaves treated with W0.2 consistently increased. Upon entering strong drought stress, the GPX activity of the soybean leaves treated with W0.2 and W1 first increased and then decreased during 6–48 h. However, the GPX activity of the soybean leaves treated with W0.5 first increased, then decreased, and then increased and was the highest among all the treatment groups at 48 h (*p* < 0.05). The GPX activity of the S-treated leaves first increased and then decreased rapidly and was lower than that of the CK leaves at 48 h (*p* < 0.05) (Figure 8B).

## 3. Discussion

Plants can improve their resistance to strong drought stress in the later stage after being subjected to a moderate drought exercise [30]. Under drought conditions, maintaining water status is a necessary requirement for ensuring the normal growth of plant leaves. Leaf wilting degree and water content are the most important drought stress indicators [31]. In the present study, soybean seedlings subjected to different drought training regimens (W0.2, W0.5, and W1) exhibited a slower decrease in water content and less leaf curling than those not subjected to drought training (S). This indicates that the drought resistance of soybean seedlings improved after weak drought treatment in the early stage. Moreover, the plants in the W0.5 treatment exhibited the greatest increase.

Weak drought can generate H_2_O_2_ signals and improve the drought resistance of plants [32]. However, H_2_O_2_ must be compared with oxidative damage markers (e.g., lipid peroxidation) for a better measure of oxidative stress [33]. We found that H_2_O_2_ accumulated in soybean leaves in vivo after 3 h under the W0.2, W0.5, and W1 treatments, while the MDA content was lower than that in the CK treatment. These findings might indicate that H_2_O_2_ plays a signaling role in vivo [34], mediating the induction of systemic acquired resistance [35]. Subsequently, the MDA content of the soybean leaves in the W0.2 and W1 treatments increased rapidly with time, showing that H_2_O_2_ acted mainly as an injury. In contrast, the MDA content was lower in the W0.5 treatment than in CK within 24 h, indicating that H_2_O_2_ in the W0.5 treatment mainly played a signaling role. Therefore, all the training treatments ultimately outperformed the S treatment. The plants in W0.5 performed best, which might be related to a special state, a pair of “parallel lines” between W0.5 and CK. The “parallel lines” indicate that the increase in the H_2_O_2_ content in the soybean leaves was basically consistent during 3–6 h. This means that the production and removal of H_2_O_2_ were balanced by the signaling effects of H_2_O_2_ accumulation, which suggests that temporary H_2_O_2_ homeostasis occurred. The W0.5 treatment produced this phenomenon at 3~6 h and 9~24 h, indicating that temporary H_2_O_2_ homeostasis can be maintained in plants under both weak and strong droughts.

H_2_O_2_ homeostasis is the balance between the production and removal of H_2_O_2_. The formation and maintenance of H_2_O_2_ homeostasis are attributed to the antioxidant system, which includes both nonenzymatic antioxidant substances and antioxidant enzymes. The concentrations of ASA and GSH change with the external environment, especially when plants are subjected to drought stress [36]. These compounds are essential for protecting cells from ROS and maintaining redox balance under drought stress [37]. Under weak osmotic stress, the increase in SOD activity continuously converted O_2_^−^ into H_2_O_2_, thereby increasing the H_2_O_2_ concentration. H_2_O_2_ accumulation in plants produced signals that induced increases in MDHAR, DHAR, GPX, GR, and APX activities and ASA and GSH content. Moreover, POD and CAT activities were also induced to scavenge excess H_2_O_2_. The increased capacity of the whole antioxidant system led to a constant increase in H_2_O_2_ content, indicating temporary H_2_O_2_ homeostasis. Under stronger drought stress, the homeostasis that occurs under weak stress is further regulated and fully functional. During the 6–9 h period, the SOD activity increased to its peak, and higher H_2_O_2_ accumulation occurred in the plants. During 9–12 h, H_2_O_2_ had the strongest effect on the contents of ASA and GSH and the activities of antioxidant enzymes, such as APX, MDHAR, DHAR, GPX, GR, SOD, POD, and CAT, to reach their peaks. Therefore, the ability to scavenge H_2_O_2_ under strong stress is higher than that under weak stress, removing more excess H_2_O_2_ and creating a higher level of homeostasis during 9–24 h.

**Figure 9:**
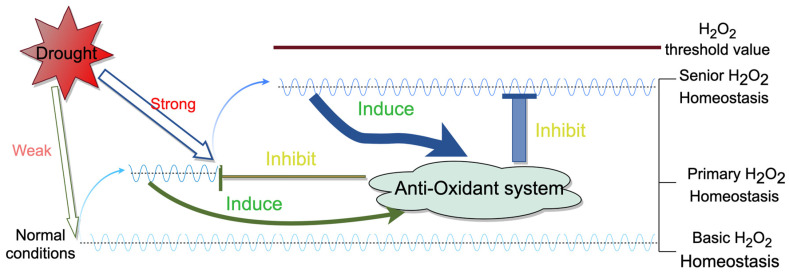
A hypothetical model for the mechanism of H_2_O_2_ homeostasis. Under normal conditions, there is basic homeostasis in the plant. Weak drought stress breaks the basic H_2_O_2_ homeostasis in soybean leaves, and the accumulation of H_2_O_2_ also induces the antioxidant system to clear excess H_2_O_2_, resulting in the formation of “primary” H_2_O_2_ homeostasis. “Primary” homeostasis also induces the formation of “senior” H_2_O_2_ homeostasis under stronger drought stresses.

Weak stress can disrupt normal homeostasis in plants, thereby generating signals [38]. When ROS levels are below a certain threshold, they can act as second messengers to transmit signals [16]. In the present study, the H_2_O_2_ content in soybean leaves under the S treatment increased more rapidly than that under the other treatments. This concentration might exceed the H_2_O_2_ threshold to induce oxidative damage, resulting in the worst plant performance. Moreover, the H_2_O_2_ content in the soybean leaves under the W0.5 treatment was lower than that under the S treatment. Taken together, these findings suggest that temporary H_2_O_2_ homeostasis might reduce H_2_O_2_ accumulation to a safe level below this threshold by further inducing antioxidant capacity. Therefore, suitable drought training could help plants adapt to stronger drought stress and maintain new H_2_O_2_ homeostasis. Under normal conditions, soybean plants are in dynamic equilibrium and have a basic H_2_O_2_ homeostasis. Soybeans under the W0.5 treatment formed primary homeostasis after entering weak stress and then advanced to senior homeostasis after entering strong stress. So, there were three H_2_O_2_ homeostasis processes in the W0.5 treatment. Based on the experimental results, we made a hypothetical model for the mechanism of H_2_O_2_ homeostasis (Figure 9). This might indicate that lower levels of H_2_O_2_ homeostasis are necessary for subsequent higher-level homeostasis, i.e., the intensity of homeostasis needs to increase stepwise. As the homeostasis level increases, plants can resist stronger stress. Under conditions of progressive drought, plants can develop higher levels of homeostasis sequentially, enabling them to tolerate stronger droughts. 

It is agreed that homeostasis exists in plants [39,40,41,42], but the mechanism of formation and function of homeostasis remain vague. In order to better study homeostasis, it is necessary to carefully select experimental conditions and develop reliable and quantitative methods [43]. We also agreed that the formation of temporary H_2_O_2_ homeostasis requires proper external conditions, such as appropriate stress intensity, temperature, humidity, and light intensity. In our experimental treatment, we specifically set up 24 h illumination to exclude the interference of the circadian clock on homeostasis [44]. Previous experiments [25,26] were carried out under normal conditions, which might account for why similar homeostasis has not been found in previous research. This special quantitatively analyzable H_2_O_2_ homeostatic state that we found in soybean leaves was a new attempt, and other types of homeostasis should also be able to achieve quantitative measurement using a corresponding special treatment.

Smith et al. [16] reported that the transcription factor CREB3 is essential for Ca^2+^, ATP, and ROS homeostasis. Yu et al. [45] found in rice that the expression of osmotic stress-related genes can be regulated by modulating ROS homeostasis. Various studies have shown that cellular homeostasis occurs as a result of the interaction of various homeostasis factors within the cell. The high performance of the W0.5 treatment group suggests that the new H_2_O_2_ homeostasis formed through the exercise can regulate the related physiological metabolism (e.g., carbon metabolism, nitrogen metabolism, and osmoregulation) to maintain homeostasis. Therefore, under the joint action of various homeostasis conditions, plants exhibit the best drought tolerance.

## 4. Materials and Methods

### 4.1. Plant Materials and Treatment

The experiment was conducted on the campus of Nanjing Agricultural University, Nanjing, Jiangsu Province, China. Uniform soybean seeds (Glycine max. cv. Kefeng 1) were selected and sown in well-drained pots (10 cm in height and 10 cm in diameter) filled with moist soil. At the 3-leaf stage, soybean seedlings exhibiting similar performance were selected and cultured in combination with Hoagland nutrient solution in a growth chamber (GXZ–800D, Jiangnan Co., Ltd., Ningbo, China). The growth conditions were as follows: 12 h light/12 h dark, a temperature of 26 °C, a luminance of 30,000 lux, and a relative humidity of 80%. After 3 days of adaptive cultivation, the seedlings were used for the experiment.

Drought stress treatment was simulated by a PEG-6000 (Jiangsu Haian Petrochemical Factory, Nantong, China). Preliminary experiments reveal that 0.2%, 0.5%, and 1% PEG-6000 caused weak stress, while 5% PEG-6000 caused strong stress on “Kefeng 1”. Therefore, 5 treatments were used in this study, and three replications were performed. The specific treatments used were as follows: Hoagland nutrient solution hydroponic mixture (CK); weak stress treatment with 0.2%, 0.5%, or 1% PEG Hoagland nutrient solution for 6 h that was then transferred to 5% PEG Hoagland nutrient solution (W0.2, W0.5, or W1); and hydroponically grown in Hoagland nutrient solution for 6 h and then transferred to 5% PEG Hoagland nutrient solution (S). Seedlings were grown under 24 h light to avoid the influence of biological rhythms on the experiment. The seedlings in each treatment group were sampled at 0, 3, 6, 9, 12, 24, and 48 h after treatment.

### 4.2. Determination of Leaf Water Content

Leaves were cut at the sampling points and quickly weighed to determine the fresh weight (Wf). Then, the leaves were first treated at 105 °C for 30 min and dried at 70 °C to a constant weight for determination of dry mass (Wd). Leaf water content = (Wf − Wd)/Wf × 100%.

### 4.3. Determination of MDA, H_2_O_2_, and O_2_^−^ Concentrations

The H_2_O_2_ concentration was measured using a commercially available kit (Jiancheng, Nanjing, China) according to the manufacturer’s instructions. The MDA content was determined by the thiobituric acid reaction, as described by Heath and Packer [46]. The rate of O_2_^−^ production was determined using the hydroxylamine method [47].

### 4.4. Determination of SOD, POD, and CAT Activities and ASA and GSH Contents

Superoxide dismutase (SOD, EC 1.15.1.1) activity was measured by the nitroblue tetrazolium (NBT) method and calculated as the ability to inhibit 50% of the total NBT reduction at 560 nm. Peroxidase (POD, EC 1.11.1.7) activity was determined by the guaiacol method, in which the change in absorbance of o-methoxyphenol at 470 nm was estimated. Catalase (CAT, EC1.11.1.6) activity was measured by the ultraviolet absorption method, in which the decrease in absorbance of H_2_O_2_ at 240 nm was estimated [48]. The ascorbic acid (ASA) and glutathione (GSH) contents were measured according to the methods of Wang et al. [49].

### 4.5. Extraction and Determination of MDHAR, DHAR, GR, APX, and GPX

The extraction and determination of monodehydroascorbate reductase (MDHAR, EC 1.6.5.4) and dehydroascorbate reductase (DHAR, EC 1.8.5.1) were performed according to the methods of Wang et al. [50]. The glutathione reductase (GR, EC 1.6.4.2) activity was determined by Loggini et al. [51]. According to the methods of Jiang and Zhang [52], ascorbate peroxidase (APX, EC 1.11.1.11) activity was determined by monitoring the oxidation rate of reduced ASA at 290 nm. Glutathione peroxidase (GPX, EC 1.11.1.9) activity was determined according to the methods of Loggini et al. [51].

### 4.6. Statistical Analysis

The experimental data were processed using Microsoft Excel 2020, and the statistical analyses were performed using SPSS 23.0 software. An ANOVA was used to evaluate significant differences among different treatments at the significance level of *p* < 0.05. Graphs were plotted using OriginPro 2020. Figure 9 was drawn using Figdraw.

## 5. Conclusions

In conclusion, the accumulation of H_2_O_2_ and the decrease in MDA in soybean plants induced by drought training indicate that H_2_O_2_ acts mainly as a signal rather than a harmful agent. The signals generated by different training intensities accordingly enhance the drought tolerance of soybean plants to different degrees. Optimal drought training further regulates the antioxidant capacity of soybean plants to remove excessive reactive oxygen species to maintain temporary H_2_O_2_ homeostasis. H_2_O_2_ homeostasis further regulates H_2_O_2_ signaling to support new homeostasis under stronger drought stress conditions, thus leading to optimal drought resistance. Plants are unable to maintain H_2_O_2_ homeostasis when they are transferred from normal conditions to strong stress conditions because the H_2_O_2_ content rapidly exceeds a certain threshold. New H_2_O_2_ homeostasis under harmful strong stress should occur progressively on the basis of previous homeostasis under unharmful mild stress.

## Data Availability

Data are contained within the article.
